# Impact of digital exposure on premarital sex and contraception use among unmarried Indian youth

**DOI:** 10.1186/s40834-024-00334-3

**Published:** 2025-01-08

**Authors:** Pragati Dattatraya Ubale, Punit Mishra, Aditi, Rajib Acharya, T.V. Sekher

**Affiliations:** 1 International Institute for Population Sciences, Mumbai, India; 2 Population Council Consulting Pvt. Ltd, New Delhi, India; 3 Population Council, New Delhi, India

**Keywords:** Adolescents, Premarital sex, PSM, Digital exposure, NFHS

## Abstract

**Background:**

Premarital sex in India is hugely stigmatized. With the widespread use of mobile phones and the internet, attitudes and behaviors towards premarital sexual activities are inevitably shifting. This study investigates the impact of digital exposure, specifically mobile phones and the internet on premarital sex and contraception use among unmarried Indian youths.

**Methodology:**

Utilizing data from the 5th National Family Health Survey, the analysis includes 172,568 women and 33,397 men aged 15–29 years. The study applies univariate, bivariate, and multivariate statistical methods, such as Chi-square tests and Multiple Logistic Regression. Propensity Score matching addresses selection bias, estimating the impact of digital exposure on premarital sexual activities and condom use.

**Results:**

The findings show that youth exposed to mobile phones and the internet are more likely to engage in premarital sex and use condoms during their first sexual encounter. Specifically, 13.46% of men and 2.83% of women reported premarital sex, with 60.84% of men using condoms at first sex. These behaviors are significantly associated with age, education, urban residence, and mass media exposure.

**Conclusion:**

Digital exposure significantly influences premarital sexual behaviors and contraception use among unmarried Indian youth. Adoption of mobile devices and internet usage in India should be accompanied by the implementation of holistic and culturally appropriate technology-driven interventions to provide sex education in India.

**Supplementary Information:**

The online version contains supplementary material available at 10.1186/s40834-024-00334-3.

## Introduction

Massive global socio-technical transformation has the potential to substantially influence human sexual behaviors, which is highly influenced by traditional norms and religious instructions. Premarital sex in India is still hugely stigmatized, undoubtedly with the widespread use of mobile phones and the internet, attitude and behavior toward premarital sexual activities are shifting. This prompts an important question: what role does technology, particularly mobile phones and the internet, play in shaping youth involvement in premarital sex? Additionally, how does it impact the use of contraceptives, especially during their first sexual encounter?

In the past decade, both national and subnational level research showed a significant proportion of youth getting involved in sex before marriage in India [1–5]. According to the 4th round of the National Family Health Survey (NFHS) conducted during the period 2015-16, 3% of never-married women and 16% of never-married men ever had sexual intercourse [4, 6]. This figure marks an increase from the corresponding figures of 0.7% & 13.5% for never-married women and men, respectively, in 2005-06 [5, 7, 8]. The National Behavioural Surveillance Survey (BSS) on sexual behaviors of youth showed that 13% of young men and 3% of young women had engaged in premarital sexual [9]. In addition, 15% of young men and 4% of young women reported experiences of premarital sex, as reported by the Youth in India survey [3]. This observed escalation could potentially be attributed to the growing liberalization of youth’s attitude towards premarital sex [10–13] and change in generations. With the mean age at marriage increasing [7, 8], the mean age at puberty decreasing [14], and the age at which mature social roles are achieved rising [15], this becomes a considerable issue. These changing norms with more normalized male-female interactions lead to widening of period for which youth are exposed to premarital sexual activities. Given, how the sexual behavior and attitudes have changed, a subsequent, equally important question is: what are the factors that are influencing this change? Researchers have identified numerous factors [1, 3, 4, 16–19] influencing the youth’s engagement in premarital sexual activities. These factors encompass various domains, including individual factors, technological factors, peer influences, family dynamics, demographic characteristics, and societal context. Individual factors such as poor academic performance, being a victim of child sexual abuse, abuse of substances, higher education, work status, being economically independent; technological factors such as access to pornographic films, mass and media, television films; peer factors such as having sexually active peers and acceptance and communication of sexual activities among peers; familial factors such as lack of parental monitoring, family instability, the strictness of parents, no communication about sex and sexuality, witnessing domestic violence; demographic factors such as migration for education and job, being a male, from urban area, declining age at puberty and increasing age at marriage; Societal factors such as deterioration of traditional and moral bonds in society, are also some of the predictor factors that many studies have discussed in consensus [1, 3, 4, 16–19].

Whenever premarital sex is discussed, the topic of contraception among unmarried youth frequently arises. This is largely because most unmarried young people are keen to avoid pregnancy before marriage, driven by the strong social stigma attached to it. Besides out-of-wedlock pregnancy, youth are also apprehensive about transmitted infections and diseases (STIs and STDs), including HIV/AIDS. Undeniably, in recent years, mobile phones and the internet have substantially facilitated the dissemination of knowledge pertaining to contraceptives, particularly condoms. Consequently, the present investigation endeavors to elucidate the influential factors, specifically mobile devices and the internet, that contribute to the utilization of condoms during individuals’ first premarital sexual encounters.

In India, there is a massive gap between awareness of condoms and their use during sexual encounters, even before marriage. According to a study on youth in India in 2011, only 5% of young women and 18% of young men who had premarital sex had used condoms at first sexual encounter [13]. According to NFHS 5 report, among the never married 15–24 youth, 1.5% of women and 7.4% of the men had sex before 12 months of the survey. Amidst them, only 62.8% of women and 61.5% of men reported using condoms during the last sexual encounter [6], which marks an increase from 36.7% of women to 51.3% of men using condoms out of 1.2 and 7.3% of sexually active women and men [7]. The reports also demonstrate that factors such as age, education, residence, caste, religion, wealth quintile, and staying away from home affect condom use among unmarried youth. In addition to that, discomfort while approaching a provider or pharmacist for condoms and having sexually active peers were inversely associated. Females were more likely to use condoms with romantic partners than non-romantic partners, and men were more likely to use condoms with non-romantic partners than romantic partners [13].

Notwithstanding, the association between technological advancement and youth sexual behavior is least explored in India. Researchers have reported that mobile phones and internet use have served as a vehicle for attaining SDG of access and effective use of contraception [20]. However, few studies confirm this association among unmarried youth. Moreover, the consensus among researchers suggests that mobile and the internet are the primary sources of sex education among youth [21]. Given the ubiquity of mobile and the internet and their wide use for sex education, it becomes imperative to comprehend the ramifications of mobile phones and internet usage on unmarried Indians’ sexual behavior as well as the use of condoms during those activities.

Social scientists have extensively demonstrated that using mass media such as newspapers, magazines, television, and radio significantly impacts sexual behaviors [4, 22]. The aforementioned sources are monological sources that give censored information without exploration. However, mobile phones and the internet are dialogical sources of information that allow exploring information through dialogues. Using mobile phones may negatively influence the youth with the internet and through exposure to sexual content such as pornographic films [23]. Mobile phones and the internet also facilitate heterosexual interactions, including sexual conversations through social media. Also, studies have shown that watching sexual content on and the internet can influence youth’s sexual behavior [22, 24, 25]. There are multiple covariates responsible for youth’s exposure to the use of the mobile phone as well as the internet among youth in itself, an exposure which is biased towards particular sections of society such as richer, well-educated, urban, economically independent, etc [6]. Hence, it is essential to reduce this selection bias of technology exposure while assessing its impact on premarital sexual behavior and condom use. A study based on UDAYA data in India has shown that exposure to mass media increases the odds of sexual intercourse as well as contraception [17]. Hence, it is vital to understand whether exposure to the internet increases the odds of youth having premarital sex and condom use. A few studies have shown that mobile phones or the internet have leveraged the premarital sexual involvement of youth. Hence, this study mainly focuses on whether digital exposure, that is, exposure to mobile and the internet, is an influencing factor for the youth’s involvement in premarital sex and protected first premarital sex using the latest Demographic and Health Surveys (DHS) dataset of India.

## Methodology

**Data Source** The study used data from the 5th round of the National Family Health Survey for the present study [6]. 

The NFHS survey used a two-stage sampling design for both rural and urban areas. The survey covered women and men aged 15–49 and 15–54 years, respectively, and gathered information on fertility, infant and child mortality, reproductive health, etc. The study employed secondary data analysis and used a sample of 172,568 women and 33,397 men between the ages of 15–29 years as per the definition of ‘Youth’ in Indian Youth Policy, 2014.

### Statistical methods

We used univariate, bivariate, and multivariate statistical analysis to elicit the results of this study. We have performed the Chi-square test and Multiple Logistic Regression to examine the association between digital exposure and other factors with the involvement in premarital sexual activities and the use of contraception at first premarital sex.

#### Dependent variables

Ever had sex (Yes/No), Used contraception at first sexual encounter (Yes/No). The information on these dependent variables was collected in the state module. Hence the sample size of these indicators related to sexual behaviors of men and women was less than that of the total sample size of the National Family Health Survey.

#### Independent variables

Mobile use, Internet use, age, the highest level of education, area of residence, caste, religion, region, substance use, working status, mobility status, mass media exposure, etc.

### Chi-square test (${\chi\:}_{c}^{2}$)

Standard Bivariate statistics were used to test whether the distribution in the categorical variables was statistically different in two or more groups.

$$\:{\chi\:}_{c}^{2}=\:\sum\:\frac{{\left({O}_{i-}{E}_{i}\right)}^{2}}{{E}_{i}}$$

Where O is the observed value, E is the expected value, and ‘i’ is the ith position in the contingency table.

### Test for multicollinearity: Pearson’s r correlation coefficient test

Test for collinearity Pearson’s r Correlation Coefficient was used to identify correlated binary, ordinal and continuous covariates. Collinearity occurs when two covariates in a multivariate model are highly related. Usually, this is because two variables represent the same thing or concept that happens simultaneously. In this analysis, mobile exposure and internet use are collinear. If we take both of them into the model, it becomes unstable. A correlation of *r* > 0.5 is often considered collinear in social sciences. When two or more covariates are collinear, we keep the one variable most strongly associated with the outcome.

### Logistic regression analysis

After applying the above tests, we scrutinized the variables, and hence after multiple logistic regression was conducted in the binary dependent variable (involvement in premarital sexual activities and use of condom at first sexual encounter) and independent variables. The logistic regression model is commonly estimated by the maximum likelihood function for dependent variables. The logistic model takes the following general form:

$$\:logit\:p=\text{ln}\left(\frac{p}{1-p}\right)={b}_{0}+{b}_{1}{x}_{1}+\:{b}_{2}{x}_{2}+\cdots\:\:{b}_{i}{x}_{i}+{e}_{i}$$

Where b_1_, b_2_, … and b_i_ represent the coefficient of each independent variable included in the model while e_i_ was an error term. Ln [p/(1-p)] represents the natural logarithms of the odds of the outcomes.

### Propensity score matching

Propensity score matching addresses selection bias and moves towards more causal estimates [26]. Propensity score matching is a quasi-experimental method of impact evaluation in observational studies where the assignment of observations into treated and control groups is not random. Selection bias is one of the primary problems faced using observational data where the assignment into treatment or control group is not random. Individuals who experience certain treatments often differ from those who don’t in systematic ways that we haven’t addressed or accounted for. We have used propensity score matching to look at the relationship between involvement in premarital sexual activities and exposure to mobile and the Internet, and we will be matching on a series of covariates such as age, education, residence, wealth index, caste, religion, mobility status, mass-media exposure, etc. In this study, we considered exposure to mobile phones and the Internet as a treatment and involvement in premarital sexual activities and using condoms at first sexual activity among men as outcome variables. Since the youth exposed to mobile phones and the internet might be significantly different from those who were unexposed, it is hard to directly compare the involvement in sex before marriage or use of condom at first sex with exposed and unexposed groups. Hence, we matched these exposed and exposed groups as much as possible and compared their outcomes. So, this matching will be done using a statistical method called propensity score matching.

The propensity score model is a binary logit/probit model with D as a dependent variable and x as an independent variable.

#### Propensity score

Propensity score is the conditional (predicted) probability that a person will receive treatment (exposure to mobile phones and exposure to the Internet) given specific pretreatment characteristics (x).

1 $$\:p\left(x\right)=p\left(D=1|x\right)=E\left(D\right|x).$$

Where, 0 < = p(x) < = 1.

D: Treatment: Exposure to mobile phones (binary variable)/ Exposure to the Internet (binary variable).

D = 1: If youth had exposure to mobile phones/internet.

D = 0: If youth did not have exposure to mobile phones/internet.

X: x is a vector of pretreatment characteristics that may affect the likelihood of being assigned to the treated group (exposed to mobile phones/internet).

Instead of matching on different x characteristics (age, education, wealth index, residence) individually, we lumped these characteristics into a propensity score and matched the observations in treated and controlled groups based on their propensity score. And once we found the matches for treated observations, next we calculated the treatment effect.

#### Calculate the treatment effect

we compared the outcome y (involvement in premarital sexual activity) between the treated and the controlled observations after matching.

y = y_1_ if D = 1: (Outcomes of the treated group- (i) Premarital sexual involvement of youth who were exposed to the mobile phones, (ii) Premarital sexual involvement of youth who were exposed to the internet, (iii) Use of contraception at the first sexual encounter by youth who were exposed to the mobile phones, (iv) Use of contraception at the first sexual encounter by youth who were exposed to the internet)

y = y_0_ if D = 0: (Outcomes of the controlled group- (i) Premarital sexual involvement of youth who were not exposed to the mobile phones, (ii) Premarital sexual involvement of youth who were not exposed to the internet, (iii) Use of contraception at the first sexual encounter by youth who were not exposed to the mobile phones, (iv) Use of contraception at the first sexual encounter by youth who were not exposed to the internet)

#### Matching method

There are several methods available for matching, such as kernel, nearest neighbor, radius, stratification, etc. Here, for this paper, we have followed a nearest neighbor matching in which we defined a common support level of 0.001 and only one nearest neighbor with replacement. For each treated observation *i*, we identified matches of controlled observation *j* with similar characteristics.

### Evaluating the impact of exposure to mobile phones/Internet on premarital sexual activities/use of condom at first sexual encounter

In PSM, three parameters were estimated. These were average treatment effect (ATE), average treatment effect among treated (ATT), and average treatment effect on untreated (ATU).

#### Average treatment effect (ATE)

It is the difference between treated and controlled observations. It is equivalent to the simple t-test between the outcome for the treated and control groups. ATE is suitable for random experiments, but it may be biased in observational studies if treated and control observations were not similar.

$$\:{\Delta\:}=\text{y}1-\text{y}0$$

$$\:ATE=E\left({\Delta\:}\right)=\text{E}\left(\text{y}1|\text{x},\:\text{D}=1\right)-\text{E}\left(\text{y}0|\text{x},\:\text{D}=0\right)$$

### Average treatment effect among treated (ATT)

$$\:ATT=E\left({y}_{1}|D=1\right)-E\left({y}_{0}\right|D=1)$$

where E (y_1_|D = 1) is the average outcome of the people who have received the treatment.

In the above equation, the second term is counterfactual: the outcome of the treated group when the group was not treated; it is not observable and needs to be estimated. This is where we can use propensity score matching. After we matched on the propensity scores, if we select those scores to be as similar as possible between the treated and the controlled group, we can just straight compare the outcomes for them.

### Average treatment effect on untreated (ATU)

$$\:ATU=E\left({y}_{1}|D=0\right)-E\left({y}_{0}\right|D=0)$$

where E $\:\left({y}_{1}|D=0\right)$ is the average observed outcome for those people who did not receive treatment. $\:E\left({y}_{0}\right|D=0)$ the counterfactual, and it shows the average outcome for those people who would have received the treatment which they had not received earlier, which is unobserved.

## Results

**Table 1:** Premarital sex among women and men aged 15–29 who had sex before marriage and condom use by men at their first sexual encounter, across selected background characteristics, India (2019-21)

Independent Variables	Women			Men					
	Sexually active women	Total number of women	Chi2 value	Sexually active men	Total number of men	Chi2 value	Men who used a condom at first sex	Number of Men	Chi2 value
**Age of the respondent**									
15–19	1.87	1,06,283	2300***	6.11	16,078	1600 ***	51.39	981	26.91***
20–24	4.15	46,450		18.96	11,315		62.32	2137	
25–29	6.1	12,406		23.05	5,839		65.4	1340	
**Highest education level**									
No education	4.97	5,281	529 ***	13.65	1,298	180***	49.09	177	84.15***
Primary	3.92	6,643		17.33	1,670		50.45	288	
Secondary	2.28	1,10,977		11.61	21,936		58.11	2539	
Higher	3.81	42,237		17.54	8,328		69.08	1455	
**Place of residence**									
Urban	3.09	57,877	6.54 **	13.4	12,409	3.67	67.81	1661	57.23***
Rural	2.69	1,07,262		13.5	20,823		56.7	2798	
**Caste**									
SC	2.93	36,779	1600***	15.74	6,470	58***	57.93	1017	44.63***
ST	4.21	15,036		17.05	2,821		46.48	479	
OBC	2.49	70,974		12.84	14,269		60.57	1829	
Other	2.81	42,349		11.81	9,672		69.95	1134	
**Religion**									
Hindu	2.75	1,31,379	2000***	14.06	25,849	97.84***	59.86	3622	11.83**
Muslim	3.12	24,716		10.29	5,584		66.32	573	
Christian	4.11	4,193		11.39	881		44.21	100	
Others	2.19	4,850		17.96	918		73.38	164	
**Wealth Quintile**									
Poorest	2.75	28,493	33.19***	14.09	5,133	6.36	47.5	723	128***
Poorer	2.83	32,587		13.52	6,618		54.74	894	
Middle	2.47	33,522		12.48	7,085		63.22	882	
Richer	2.63	34,710		13.13	7,478		63.49	976	
Richest	3.4	35,827		14.3	6,917		71.43	984	
**Region**									
North	2.81	26,214	2300***	16.26	3,045	333***	69.16	492	87.76***
Central	4.35	50,511		18.92	4,022		52.96	759	
East	2.71	33,095		13.4	8,292		55.77	1106	
Northeast	3.32	5,707		8.32	1,686		56	139	
West	2.14	21,185		15.81	8,025		74.17	1269	
South	0.68	28,427		8.54	8,163		48.26	694	
**Drinks Alcohol**									
No	2.76	1,64,596	2800***	10.77	29,288	1400***	62.31	3148	16.01***
Yes	23.35	543		33.48	3,944		57.32	1310	
**Ever worked**									
No	2.67	19,584	53.20***	7.29	16,218	894***	62.22	1180	25.08***
Yes	4.2	4,857		19.34	17,014		60.34	3278	
**Away from home for more than one month in the past 12 months**									
No	2.72	22,921	44.01***	12.3	28,279		61.13	3467	0.06
Yes	6.79	1,519		20.07	4,953	320 ***	59.82	991	
**Has a bank or saving account that they themselves use**									
No	2.07	6,721	24.67***	11.05	5,297	168 ***	38.65	584	113.67***
Yes	3.32	17,719		17.23	19,167		63.86	3289	
**Has a mobile phone they themselves use**									
No	1.77	14,093	264 ***	5.54	2,851	463***	38.06	157	27.34***
Yes	4.62	10,348		17.26	21,613		60.98	3716	
**Ever used Internet**									
No	2.41	12,048	65.07***	12.32	5,921	173.36***	49.91	730	46.39***
Yes	3.52	12,392		17.03	18,543		62.41	3144	
**Can read SMS**									
No	2.52	3,735	15.28***						
Yes	3.02	20,313							
**Mass media exposure**									
No	2.76	25,737	31.70***	11.11	3,031	10.62**	49.44	331	30.66***
Partial	2.71	1,20,477		14	23,627		61.07	3302	
Full	3.64	18,926		12.63	6,574		64.49	826	
**Total**	**2.83**	**1**,**65**,**139**		**13.46**	**33**,**232**		**60.84**	**4459**	
	**4**,**668**			**4**,**474**					

***p < 0.01, **p < 0.05, *p < 0.1

**Table 2A:** Unadjusted odds ratio and 95% CI for mobile phones association with premarital sex of women and men and condom use at the first sexual encounter in India (2019-21)

	Premarital sex among women		Premarital sex among men		Condom use at first sexual encounter among men	
Ever had sex	Odds Ratio	[95% CI]	Odds Ratio	[95% CI]	Odds Ratio	[95% CI]
**Mobile use**						
No (ref.)						
Yes	3.18***	[2.75, 3.69]	4.29***	[3.72, 4.96]	2.10***	[1.58, 2.79]
_cons	0.02	[0.02, 0.02]	0.05	[0.05, 0.06]	0.7	[0.53, 0.93]
Observations	25,877		27,904		4,630	

* *p* < 0.1, ** *p* < 0.05, *** *p* < 0.01; (ref.) represents the reference category

**Table 2B:** Unadjusted odds ratio and 95% CI for internet use association with premarital sex of women and men and condom use at the first sexual encounter in India (2019-21)

	Premarital sex among women		Premarital sex among men		Condom use at first sexual encounter among men	
Ever had sex	Odds Ratio	[95% CI]	Odds Ratio	[95% CI]	Odds Ratio	[95% CI]
**Internet use**						
No (ref.)						
Yes	1.75***	[1.53, 2.01]	1.70***	[1.58, 1.85]	1.68***	[1.45, 1.96]
_cons	0.03	[0.02, 0.03]	0.13	[0.12, 0.14]	0.93	[0.81, 1.07]
Observations	25,877		27,904		4,630	

* *p* < 0.1, ** *p* < 0.05, *** *p* < 0.01; (ref.) represents the reference category

The chi-square test for two dependent variables, premarital sexual involvement of unmarried women and men, and use of condom by men at first premarital sexual encounter against a series of independent variables is illustrated in Table 1 (in the Appendix). From the sample of 172,568 unmarried women and 33,397 unmarried men, 2.83% of women and 13.46% of men reported their involvement in sex prior to marriage. However, 60.84% of sexually active unmarried men reported the use of condom at their first premarital sexual encounter. The table did not show the women’s reporting of use of condom at first sexual encounter as there were only 713 women who responded to this question. Out of these 713 never married women, who ever had sex, 51.8% (*n* = 369) reported use of condom at first sexual encounter. When compared across different caste categories, both women and men from the ST category were more likely to report engagement in premarital sex, and men were least likely to use condoms than any other caste category. Individuals from the middle wealth quintile displayed the least percentage of premarital sex (2.47% women and 12.48% men). However, there was a decline in the percentage of men reporting the use of condom at first sexual encounter as we transitioned from richest to poorest wealth index. Central Indian youth had the highest prevalence of premarital sex (4.35% women, 18.92% men), while those from the southern region had the lowest (0.68% women, 8.54% men). However, men from the south had the lowest reported percentage, followed by men from the central region when it came to using condoms during their first sexual encounter. Alcohol drinkers had higher rates of premarital sex than non-drinkers. Among women, 23.35% of drinkers and 2.76% of non-drinkers engaged in premarital sex. For men, the rates were 33.48% for drinkers and 10.77% for non-drinkers. The usage of mobile phones and the Internet exhibited a positive association with the involvement of both women and men in premarital sexual activity, as well as their adoption of condom usage during their initial premarital sexual encounters. The youth who used mobile phones, among them 4.62% of women and 17.26% of men reported premarital sexual involvement compared to 1.77% of women and 5.54% of men who did not use mobile. Moreover, among women and men who used the internet, 3.52% of women and 17.03% of men reported premarital sex, while the percentage was 2.41% for women and 12.32% for non-internet users. Women and men who were fully exposed to mass media (TV, Radio, and newspaper) were more involved in premarital sex (3.64% women, 12.63% men) than unexposed (2.76% women and 11.11% men), and men were more likely to report condom use when exposed to mass media (64.49% fully exposed to mass media versus 49.44% unexposed).

Highly significant outcomes of an unadjusted logistic regression analysis wherein the dependent variable was “ever had premarital sex,” and “Use of condom at first sexual encounter” the independent variables were “use mobile phone” and “use of the internet” are shown in Tables 2A and 2B. The odds ratio estimates indicated that women who used a mobile phone had 3.18 times higher odds of ever having sex than those who never used a mobile phone. Similarly, men who used a mobile phone had 4.29 times higher odds of ever having sex compared to those who did not use a mobile phone. The 95% confidence interval for the odds ratio ranges from 2.75 to 3.69 for the women’s sample and 3.72 to 4.96 for the men’s sample. The odds of using condoms at first sexual encounter increase to 2.10 if men use mobile phones. Similarly, if exposed to the internet, women had 1.75 times higher odds, and men had 1.70 times higher odds of having sex before marriage. The odds of using condoms at first sexual encounters increased to 1.68 times if men used the internet.

To ascertain the significance of mobile phone use as an influencer in youth’s involvement in premarital sex as well as the use of condoms at first sex in the presence of multiple other independent variables, we conducted an adjusted logistic regression analysis. Table 3 represented three statistically significant models of multiple logistic regression; one was premarital sexual involvement of women (*n* = 25877; LR chi sq = 887.27), second was premarital sexual involvement of men (*n* = 27904; LR chi sq = 2975.64) and third was the use of a condom at a first sexual encounter by men (*n* = 4630; LR chi sq = 305.96). The likelihood ratio chi-square tests indicates that the models were statistically significant (*p* < 0.001), suggesting that the predictors included in the model were related to the outcome. The odds of getting involved in premarital sex by women and men and using condoms at first sex by unmarried men decreased from unadjusted to adjusted logistic regression. In multiple logistic regression, the odds of women and men’s involvement in premarital sex were 2.30 times higher if they used mobile than their counterparts. Furthermore, men who use mobile were 42% more likely to use condom at first sex than those who did not use mobile phones. The magnitude of the odds ratio represented the positive relationship between the predictor variable age and the dependent variable premarital sex. The likelihood of women engaging in premarital sex in the age group 20–24 was 1.85 times, and in the age group 25–29 was 2.60 times higher than women in the age group 15–19. Similarly, men of the 20–24 age group were 2.20 times, and of 25–29 were 2.53 times highly likely to get involved in premarital sex than that of 15–19 years. The likelihood of women’s involvement in sex before marriage was higher for the uneducated than the educated youth. The odds ratio of 0.54 & 0.54 indicates that higher-educated and secondary-educated women have approximately half the likelihood of being involved in premarital sex compared to uneducated women. Men from rural areas were 10% more likely to have premarital sex and 25% less likely to use condom at first sexual encounter than urban men. Women and men belonging to the Scheduled Tribe category were 24% and 12% more likely to involve in premarital sex than the SC caste. Christian women had 2.26 times higher odds of experiencing premarital sex than Hindu women. Men who identified themselves as Christian were 33% more likely to report premarital sexual activity and 36% less likely to report use of condom at sexual debut before marriage. Multiple logistic regression did not show a significant association between sex before marriage and the wealth quintile of the respondent. However, the richest men were 41% significantly more likely to use condom at their first sexual debut before marriage than the poorest.

**Table 3:** Adjusted odds ratio and 95% CI for mobile phones’ usage association with premarital sex of women and men and condom use at the first sexual encounter in India (2019-21)

Independent Variables	Categories	Premarital sex among women		Premarital sex among men		Condom use at first sexual encounter among men	
		OR	95% CI	OR	95% CI	OR	95% CI
**Mobile use**	No (ref.)	1	[1, 1]	1	[1, 1]	1	[1, 1]
	Yes	2.30***	[1.93, 2.74]	2.34***	[2, 2.73]	1.42**	[1.05, 1.91]
**Age**	15–19 ﻿(ref.)	1	[1, 1]	1	[1, 1]	1	[1, 1]
	20–24	1.85***	[1.56, 2.2]	2.20***	[2.01, 2.4]	1.01	[0.86, 1.18]
	25–29	2.60***	[2.1, 3.22]	2.53***	[2.29, 2.81]	1.16	[0.96, 1.4]
**Highest level of education**	No education ﻿(ref.)	1	[1, 1]	1	[1, 1]	1	[1, 1]
	Primary	0.85	[0.55, 1.31]	1.02	[0.83, 1.26]	1.06	[0.73, 1.54]
	Secondary	0.54***	[0.39, 0.75]	0.92	[0.77, 1.09]	0.97	[0.71, 1.31]
	Higher	0.53***	[0.37, 0.75]	0.96	[0.8, 1.15]	1.19	[0.85, 1.66]
**Area of residence**	Urban ﻿(ref.)	1	[1, 1]	1	[1, 1]	1	[1, 1]
	Rural	0.89	[0.74, 1.05]	1.10**	[1.01, 1.2]	0.75***	[0.63, 0.88]
**Caste**	SC ﻿(ref.)	1	[1, 1]	1	[1, 1]	1	[1, 1]
	ST	1.24*	[0.97, 1.58]	1.12*	[0.99, 1.26]	0.92	[0.75, 1.15]
	OBC	0.88	[0.71, 1.09]	1.00	[0.91, 1.1]	1.08	[0.91, 1.28]
	None of the above	0.70***	[0.55, 0.9]	0.77***	[0.69, 0.86]	1.08	[0.88, 1.31]
**Religion**	Hindu ﻿(ref.)	1	[1, 1]	1	[1, 1]	1	[1, 1]
	Muslim	1.11	[0.88, 1.41]	0.91	[0.81, 1.02]	1.09	[0.87, 1.37]
	Christian	2.26***	[1.73, 2.95]	1.33***	[1.12, 1.58]	0.64***	[0.47, 0.89]
	Others	1.41*	[1.06, 1.87]	1.27***	[1.1, 1.47]	0.84	[0.65, 1.1]
**Wealth quintile**	Poorest ﻿(ref.)	1	[1, 1]	1	[1, 1]	1	[1, 1]
	Poorer	1.05	[0.85, 1.31]	1.06	[0.95, 1.18]	1.03	[0.85, 1.25]
	Middle	1.02	[0.8, 1.29]	0.97	[0.86, 1.09]	1.17	[0.95, 1.44]
	Richer	0.90	[0.69, 1.17]	1.03	[0.91, 1.17]	1.15	[0.92, 1.44]
	Richest	0.95	[0.71, 1.28]	1.04	[0.9, 1.2]	1.41***	[1.09, 1.83]
**Region**	North ﻿(ref.)	1	[1, 1]	1	[1, 1]	1	[1, 1]
	Central	2.30***	[1.83, 2.9]	1.27***	[1.15, 1.4]	0.63***	[0.52, 0.75]
	East	1.39**	[1.05, 1.83]	0.63***	[0.56, 0.72]	0.69***	[0.55, 0.88]
	Northeast	1.37**	[1.03, 1.83]	0.59***	[0.51, 0.68]	1.05	[0.79, 1.4]
	West	1.49***	[1.11, 2]	1.15**	[1.02, 1.31]	0.87	[0.69, 1.11]
	South	0.30***	[0.19, 0.47]	0.29***	[0.25, 0.33]	0.66***	[0.5, 0.86]
**Drinks Alcohol**	No ﻿(ref.)	1	[1, 1]	1	[1, 1]	1	[1, 1]
	Yes	5.26***	[3.7, 7.48]	3.02***	[2.78, 3.29]	0.85**	[0.73, 0.97]
**Ever worked**	Never ﻿(ref.)	1	[1, 1]	1	[1, 1]	1	[1, 1]
	Yes	1.10	[0.94, 1.29]	1.50***	[1.38, 1.63]	0.79***	[0.67, 0.92]
**Stayed away from home**	No ﻿(ref.)	1	[1, 1]	1	[1, 1]	1	[1, 1]
	Yes	1.52***	[1.22, 1.89]	1.44***	[1.32, 1.56]	1.14*	[0.99, 1.32]
**Bank account**	No ﻿(ref.)	1	[1, 1]	1	[1, 1]	1	[1, 1]
	Yes	1.19*	[0.99, 1.42]	1.32***	[1.2, 1.46]	1.92***	[1.6, 2.29]
**Mass-media exposure**	No ﻿(ref.)	1	[1, 1]	1	[1, 1]	1	[1, 1]
	Partial	1.16	[0.94, 1.43]	1.16**	[1.03, 1.29]	1.25**	[1.02, 1.54]
	Full	1.31	[1, 1.71]	1.27***	[1.11, 1.45]	1.27*	[1, 1.61]
Constant		0.01	[0.01, 0.02]	0.03	[0.02, 0.03]	0.77	[0.47, 1.26]
**Observations**		25,877		27,904		4630	

* *p* < 0.1, ** *p* < 0.05, *** *p* < 0.01; (ref.) represents the reference category

Note - _cons refers to the intercept (also known as the constant term) of the model. It represents the log-odds of the dependent variable (in this case, premarital sex of youth) when all the independent variables in the model are set to zero

The odds of women and men who reported positive status of drinking alcohol were 5.26 times and 3.02 times higher to engage in premarital sex than those who did not drink. However, men who drink were 15% less likely to use condom at the first sexual encounter. Youth who spent a minimum of one month away from their residence within the preceding 12 months demonstrated a higher likelihood to engage in sexual intercourse before marriage, as well as a greater propensity of using a condom during their initial sexual encounter when compared to those who did not have experienced time away from home. Having bank accounts that youth themselves use had highly positively associated with their involvement in sex before marriage and use of condom at first sex. Women and men with bank accounts were 19% and 32% more likely to engage in sex than their counterparts. Notably, men with bank accounts were 92% more likely to use condom on their first sex before marriage. Compared to men with no exposure to TV, radio, or newspapers, those with partial and complete exposure were 16% and 27% more likely to indulge in sexual intercourse before marriage and 25% and 27% more likely to engage in first sex by using condom.

In the present paper, we have not shown the adjusted logistic regression for the association between engagement in premarital sex and internet exposure and use of a condom at first sexual encounter and the internet exposure with multiple other predictor variables, but the internet use remained a statistically significant predictor of premarital sexual involvement as well as use of a condom at first sexual encounter after adjusting for other independent variables as well.

Despite the positive association between using mobile phones and the internet and engaging in sexual relationships prior to marriage and the use of condom at the first sexual encounter, it’s essential to recognize that multiple factors can influence the decision to use or not use mobile or internet. Therefore, there is a potential for selection bias in the obtained results. To address this concern, we employed propensity score matching to mitigate the bias in treatment assignment or mobile phone use.

Table 6 (in the *Additional file 1*) showed significant associations between treatment (exposure to mobile phones) and a set of independent variables. These variables were associated with the youth’s propensity to use mobile phones and could potentially confound the estimation of the effect of mobile phones on involvement in premarital sex as well as the use of condom at premarital sexual debut. In other words, our results showed that mobile usage was associated with premarital sexual engagement and the use of condom in those activities. However, the treated (exposed to mobile) and untreated (unexposed to mobile) groups differed on many grounds. Consequently, we employed propensity score matching to mitigate the bias in treatment assignment and accurately estimate the impact of mobile phone usage on the sexual behaviors of youth. In the presented study, we did not show the association between internet use and socio-demographic and economic characteristics, but there was a significant association.

**Table 4:** The effect of mobile exposure on youth’s involvement in premarital sex and their use of condom at first sexual encounter, analysis using propensity score matching, India (2019-21)

Variables	The effect of mobile exposure on sexual involvement among unmarried women						Common support			
	Sample	Treated	Controls	Difference	S.E.	T-stat		Off support	On support	Total
Ever had sex (Women)	Unmatched	0.056	0.018	0.037	0.002	16.35	Untreated	46	13,961	14,007
	ATT	0.054	0.028	0.026	0.006	4.17	Treated	96	11,774	11,870
	ATU	0.018	0.05	0.032	.	.	Total	142	25,735	25,877
	ATE			0.029	.	.				
	**The effect of mobile exposure on sexual involvement among unmarried men**						**Common support**			
Ever had sex (Men)	Unmatched	0.187	0.051	0.136	0.006	21.7		Off support	On support	Total
	ATT	0.187	0.143	0.044	0.015	2.9	Untreated	0	4,074	4,074
	ATU	0.051	0.126	0.075	.	.	Treated	45	23,785	23,830
	ATE			0.048	.	.	Total	45	27,859	27,904
	**The effect of mobile exposure on the use of condom at first sexual involvement among unmarried men**						**Common support**			
Condom use at the first sexual encounter before marriage (Men)	Unmatched	0.596	0.413	0.183	0.035	5.24		Off support	On support	Total
	ATT	0.589	0.39	0.199	0.062	3.22	Untreated	1	205	206
	ATU	0.415	0.541	0.127	.	.	Treated	202	4,222	4,424
	ATE			0.196	.	.	Total	203	4,427	4,630

The results obtained using propensity score matching to understand the impact of mobile phones on youth’s involvement in premarital sex and the use of condoms at first sexual encounters can be seen in Table 4. PSM eliminates most of the bias attributable to observable covariates. The final number of blocks for the women’s sample was 14 and men’s 17. The unmatched sample estimate presents the raw estimates, i.e., without matching, the result showed that for those who had exposure to mobile phones, women had 3.7% higher chances, and men had 13.6% higher chances to get involved in premarital sex than those who did not receive the exposure of mobile phones. ATT, ATU, and ATE show the estimates after nearest neighbor matching with replacement. For women and men, the average treatment effect on the treated (ATT) values among treated and controls were 0.054 & 0.028 and 0.187 & 0.143, respectively, which means that if the mobile exposure was not received by those youth who received it, the prevalence of premarital sex would have been 2.8% and 14.3% for women and men respectively. ATU values in treated and control groups for women were 0.018 and 0.050, respectively, and for men, were 0.051 and 0.126, which means among women and men who did not have mobile exposure, their chance of having premarital sex will increase by 3.2% and 7.5% respectively if they would have received mobile exposure. Average treatment effect (ATE) showed the difference between the treated and the untreated, which was 0.029 and 0.048 for women and men, respectively, which means, on average, there was a 2.9% and 4.8% higher chance of involving in premarital sex for women and men respectively who were exposed to mobile phones.

Similarly, in the case of the impact of mobile phones on men’s use of contraception, when unmatched, the men who were exposed to mobile use had 18.3% higher chances of using contraception at their first sexual encounter. For men, the average treatment effect on the treated (ATT) values among treated and controls were 0.589 & 0.390, which means that if the mobile exposure were not received by those men who received it, the prevalence of using contraception at first sexual encounter would have been 39%. ATU values in treated and control groups were 0.415 and 0.541, respectively, which means among men who did not have mobile exposure, their chance of using a condom at first sex will increase by 12.7% if they would have received mobile exposure. Moreover, the ATE value indicates that, on average, there were 19.6% higher chances of using condom at first sex for unmarried men who were exposed to mobile phones than that of unexposed.

When compared Unmatched and ATE values for difference, it can be concluded that the after matching the difference between exposed and exposed groups to mobile, involvement in premarital sex has decreased and increased when considered condom use at first sexual encounter.

**Table 5:** The effect of internet exposure on youth’s involvement in premarital sex and their use of condom at first sexual encounter, analysis using propensity score matching, India (2019-21)

Variable	The effect of Internet exposure on sexual involvement among unmarried women						Common support			
	Sample	Treated	Controls	Difference	S.E.	T-stat		Off support	On support	Total
Ever had sex	Unmatched	0.044	0.026	0.019	0.002	8.08	Untreated	74	12,460	12,534
	ATT	0.044	0.033	0.011	0.007	1.54	Treated	43	13,300	13,343
	ATU	0.026	0.034	0.008	.	.	Total	117	25,760	25,877
	ATE			0.01	.	.				
	**The effect of the Internet exposure on sexual involvement among unmarried men**						**Common support**			
Ever had sex	Unmatched	0.184	0.117	0.067	0.005	13.21		Off support	On support	Total
	ATT	0.184	0.152	0.032	0.014	2.33	Untreated	8	7,121	7,129
	ATU	0.117	0.17	0.053	.	.	Treated	286	20,489	20,775
	ATE			0.038	.	.	Total	294	27,610	27,904
	**The effect of the Internet exposure on use of condom at first sexual involvement among unmarried men**						**Common support**			
Condom use at the first sexual encounter before marriage	Unmatched	0.611	0.483	0.128	0.019	6.84		Off support	On support	Total
	ATT	0.607	0.525	0.082	0.04	2.09	Untreated	1	830	831
	ATU	0.483	0.587	0.104	.	.	Treated	102	3,697	3,799
	ATE			0.086			Total	103	4,527	4,630

Table 5 represents results obtained using propensity score matching to understand the impact of internet exposure on youth’s involvement in premarital sex and the use of condoms at first sexual encounters. The final number of blocks for women’s and men’s samples was 15. The unmatched sample estimate in Table 5 presents the raw estimates, i.e., without matching, the result showed that for those who had exposure to the Internet, women had 1.9% higher chances, and men had 6.7% higher chances to get involved in premarital sex than those who did not receive the exposure of the Internet. For women and men, the average treatment effect on the treated (ATT) values among treated and controls were 0.044 & 0.033 and 0.184 & 0.152, respectively, which means that if the Internet exposure were not received by those youth who received it, the prevalence of premarital sex would have been 3.3% and 15.2% for women and men respectively. ATU values in treated and control groups for internet exposure were 0.026 and 0.034, respectively, for women and 0.117 and 0.170 for men, which means youth who were not exposed to the Internet if they would have used it, their chance of having sex before marriage will increase by 0.8% and 5.3% respectively. ATE of 0.01 and 0.038 for women and men, respectively, which means, on average, there was a 1% and 3.8% higher chance of involving in premarital sex for women and men respectively who were exposed to internet.

Similarly, in case, if the impact of internet use on men’s use of contraception, when unmatched the men who were exposed to internet use had 12.8% higher chances of using contraception at their first sexual encounter. For men, the average treatment effect on the treated (ATT) values among treated and controls were 0.607 & 0.525, which means that if the internet exposure was not received by those men who received it, the prevalence of using contraception at first sexual encounter would have been 52.5%. ATU values in treated and control groups were 0.483 and 0.587, respectively, which means among men who did not have internet exposure, their chance of using a condom at first sex will increase by 10.4% if they would have received internet exposure. Moreover, the ATE value indicated that, on average, there was an 8.6% higher chance of using a condom at first sex for unmarried men exposed to the internet than that of unexposed.

**Fig. 1:**
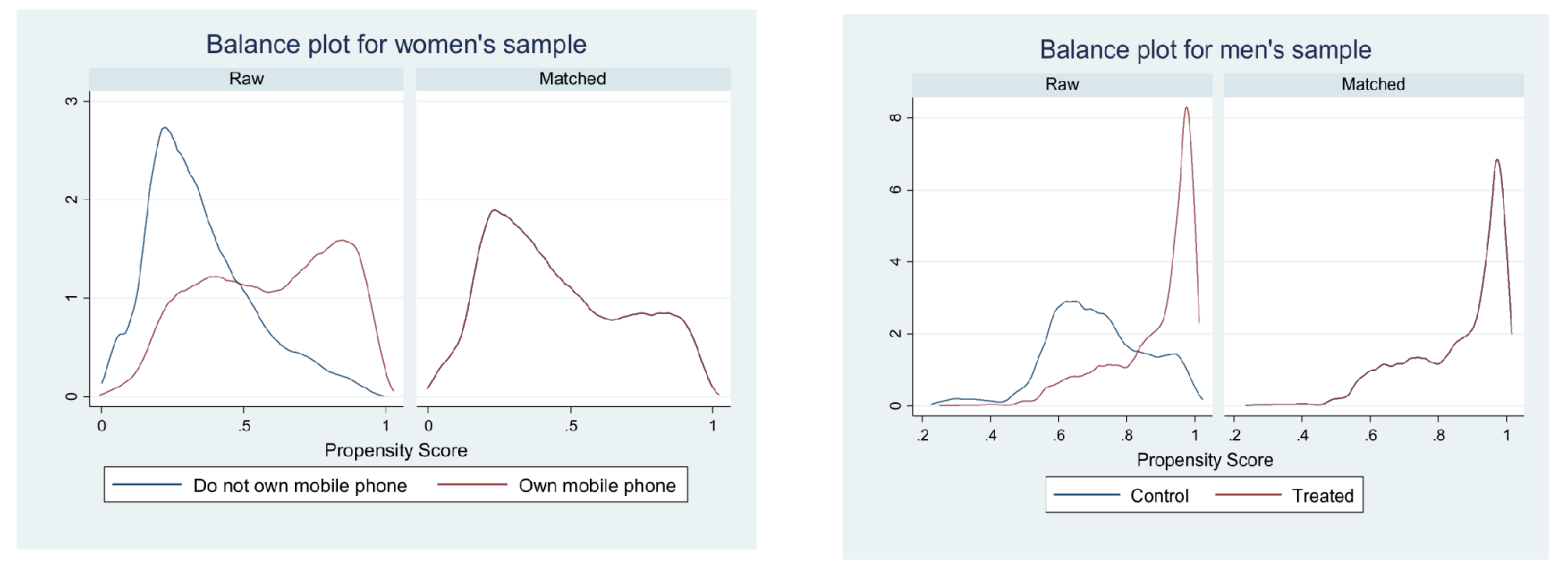
Balance plots for treatment: mobile exposure

**Fig. 2:**
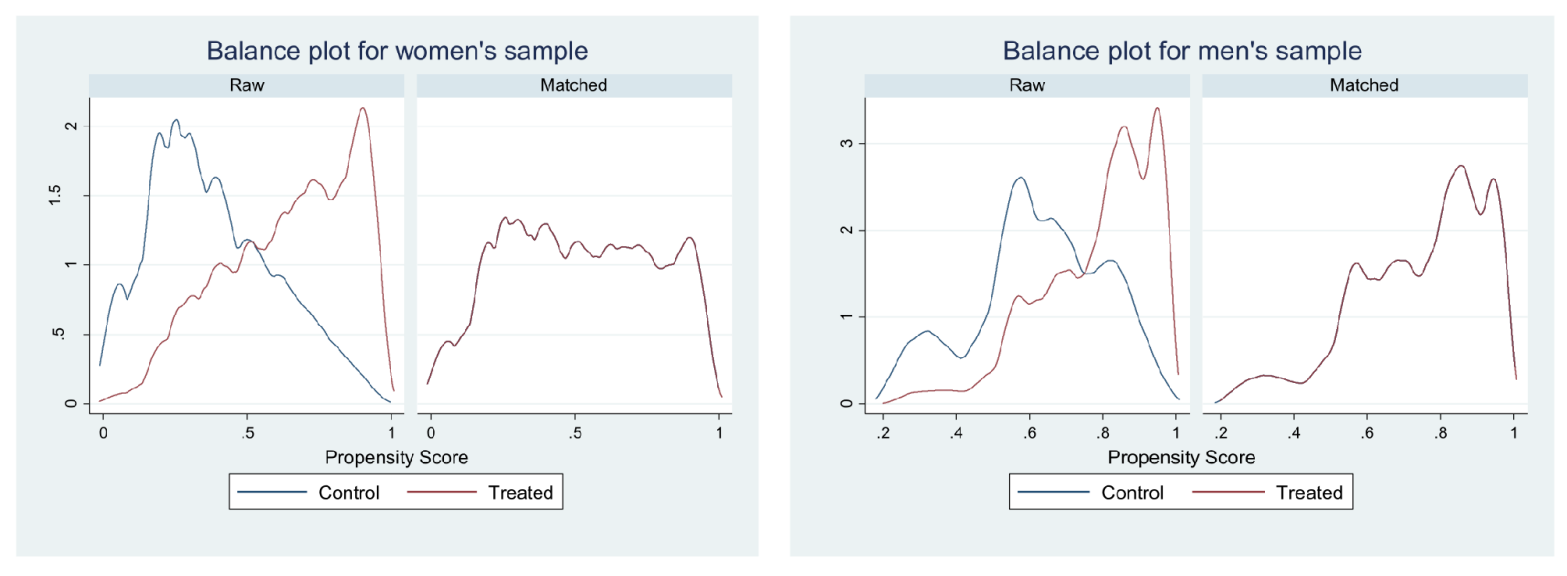
Balance plots for treatment: internet exposure

**Fig. 3:**
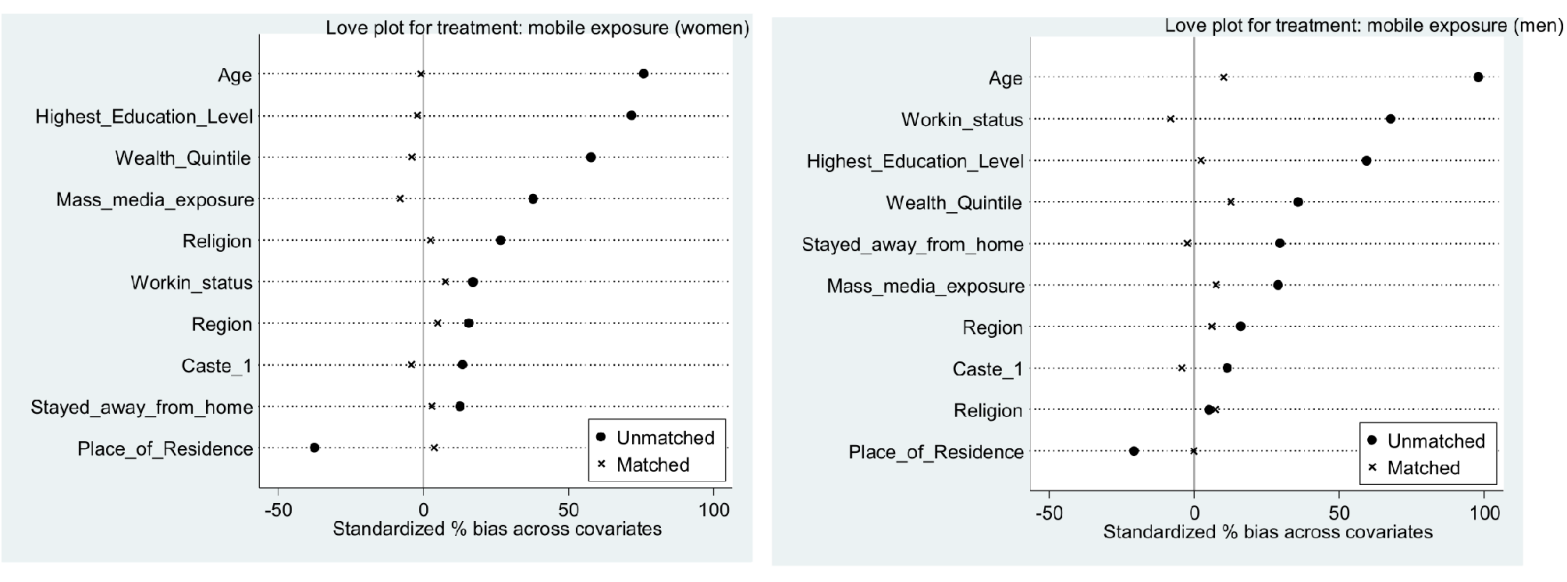
Love plots for treatment: mobile exposure

**Fig. 4:**
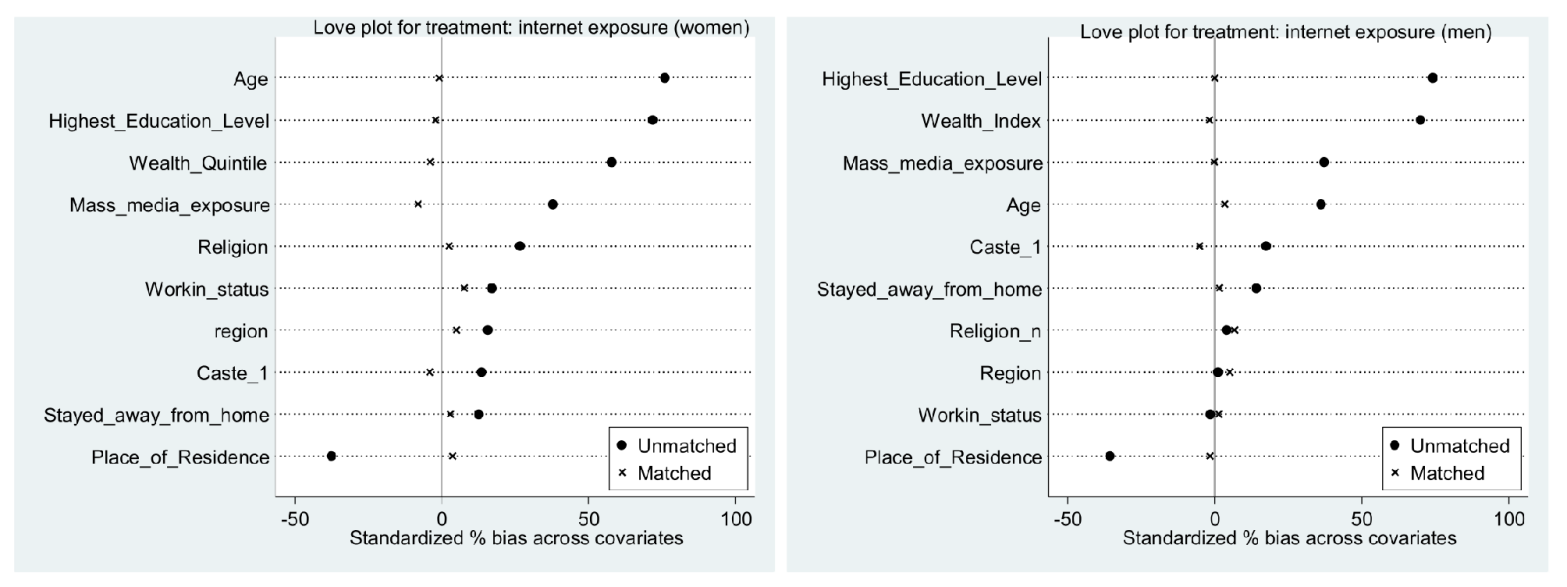
Love plots for treatment: internet exposure

When compared Unmatched and ATE values for difference, it can be concluded that the after matching the difference between exposed and exposed groups to the internet, involvement in premarital sex and condom use at first sexual encounter has decreased. Tables 4 and 5 also showed the number of dropped observation due to common support were minimal in all six cases. The balance plot of the covariates of the treatment and control group for mobile exposure and internet exposure before and after matching cases has been shown in Figs. 1 and 2, respectively. It indicates that both the control and treatment groups were balanced, indicating unbiasedness in the estimated treatment effects. Love plot showed standardized % bias across all covariates in matched and unmatched samples (Figs. 3 and 4).

## Discussion

The findings from the current study demonstrate that the youth are breaking the traditional norms about keeping celibacy until marriage and their involvement in premarital sex for men (13.46%) seems relatively more common (five-times more) than for women (2.83%). These findings are in alignment with prior research, where the percentage of men’s participation in sex prior to marriage is almost five times higher (13–16%) than that of women (1–3%) [3–6, 8, 9]. This gender difference could be attributed to two factors, sexual double standards as well as social desirability bias. In India, women’s chastity is more highly valued when it comes to marriage than men’s, leading to lower sexual involvement of women prior to marriage and relatively more laxity to men for sexual involvement. Additionally, because of the higher value on chastity and the stigma of women’s participation in sex before marriage, women can be more likely to underreport their sexual involvements. Around, 60.84% of men who ever had sex reported that they used condom at their first sexual encounter. Which reveals that significant proportion (40%) of men were exposed to the risk of unwanted pregnancy, STIs/STDs and possible unsafe abortions of their partners.

This study provided evidence that individuals belonging to certain demographic groups, such as older age, uneducated, having high education level, urban resident, ST category, Christian women, Hindu men, belonging to the richest wealth quintile, residing in the central region, consuming alcohol, having work experience, being away from home, and having full exposure to media, were more inclined to report engaging in premarital sexual activity compared to their counterparts in the respective categories. Youths of age (15–19), who had lower levels of education, resided in rural areas, belonged to the scheduled tribe (ST) category, followed the Christian religion, fell within the poorest wealth quintile, lived in the southern region, engaged in alcohol consumption, had work experience, lived away from home, did not possess a bank account, and had no exposure to mass media exhibited the lowest likelihood of utilizing condoms during their first premarital sexual encounter. When comparing demographic factors, it was found that young individuals who lacked formal education, belonged to the Scheduled Tribe (ST) category, identified with the Christian religion, consumed alcohol, and had work experience, displayed a twofold vulnerability as they exhibited a higher propensity for engaging in premarital sexual activity and were also less likely to use condoms at their first sexual encounter.

Central Indian women as well as men reported the highest prevalence of involvement in premarital sex, while Central Indian men have the second lowest rates of condom use at first sexual encounter after Southern men. This could be because the state of Chhattisgarh and Madhya Pradesh are predominantly tribal. Previous studies have also discussed that youth belonging to Scheduled Tribes have the highest rates of premarital sex [4, 27], possibly because of more permissive cultural attitudes towards it [28]. Additionally, the lowest condom use is probably because of a younger age at sexual debut [4].

The key objective of this investigation was to unravel the ramifications of digital exposure on the engagement in premarital sexual activity and the utilization of condoms at first sex before marriage. All the analytical methodologies employed, including the Chi-square test, unadjusted logistic regression, adjusted logistic regression, and propensity score matching, show that there was a positive association between the use of mobile and internet with higher premarital sexual involvement of youth and elevated contraception use at the first sexual encounter. PSM produced evidence that the after matching the difference in involvement in premarital sex between the youth who had exposure to mobile and who had not was reduced to a greater extent. However, all the aforementioned methodologies produced results that exposure to mobile and internet was a strong predictor of use of condom at first premarital sexual encounter by unmarried men. PSM showed evidence that after-matching the difference in the use of contraception between the youth who had exposure to mobile/internet and those who had not, has increased, indicating exposure contributed positively to condom use at first sex. In addition, a positive association existed between women’s ability to read SMSs and their increased likelihood of having sex before marriage. Studies showed that mobile or internet exposure is associated with increased knowledge of premarital sex and contraception use among youth [29]. Numerous studies conducted in India reported that in the absence of a credible person or authenticated source of knowledge for sex education, youth increasingly resort to the internet [21, 30]. Regrettably, the unsupervised internet usage also leads youth to pornographic sites or other sexual content.

The authors of this study posit the possibility that the inclination to premarital sexual participation among unmarried Indian adolescents is affected not exclusively by the usage of mobile devices or internet connectivity but rather by social media, heterosexual engagement, and exposure to sexual content facilitated by the unsupervised use of the internet and mobile. For instance, significantly fewer studies show that mobile use or the internet causes increased premarital sexual involvement. But exposure to sexual content such as pornography was cited as one of the significant influencing factors for youth to get involved in premarital sex in number of studies [31]. A Vietnamese study showed that exposure to porn or sexual media was a highly influencing factor for premarital sexual involvement [32]. In another study, around 70% of sexually active adolescents had viewed pornography, with the internet (59%) being the primary source, followed by videos (19%) and mobile telephones (14%) [33]. Moreover, some studies reported a positive association between the internet and sexual activities before marriage, but the association was inconsistent. A study by Kumar et al. in 2013 found a positive association between boys’ exposure to the internet and higher involvement in premarital sex, while no such association was found among men [19].

Though the current study reported that mobile and internet exposure plays an important role of being a predictor of youth’s involvement in premarital sexual involvement, consistent with previous studies, our results also showed that these play a significant role in making sexual encounters with protection [29]. Systematic reviews, including reviews of randomized control trials, have shown that the use of mobile phones to deliver information on sex and sexuality has not only just improved the number of safe sexual activities but also promoted the utilization of health services such as STI testing and improved health outcomes among young people [16, 31, 34, 35]. Digital media can overcome barriers to adolescent or youth health care by reducing expenses, long waiting times, stigma, provider biases, and fear of the absence of privacy or confidentiality [16]. The use of mobile phones, the internet, and other digital media in the context of healthcare is commonly referred to as mHealth, which stands for mobile health [16]; mHealth has gained significant attention in recent years due to the ubiquitous availability of mobile devices and accessibility to the internet and their potential to improve healthcare accessibility, efficiency, and patient engagement.

The PSM analysis indicated that a higher percentage of both women and men were engaged in premarital sex when exposed to digital media; however, this increase is more pronounced among men than women. Furthermore, bivariate analysis revealed that the urban women were more likely to engage in premarital sex compared to their rural counterparts, and this difference was not significant among men. This could be attributable to gender as well as rural-urban divide in digital exposure [36] in India. Although the gender gap narrowed in 2023 in India, only a one-third (35%) of women compared to half of men (51%) own a smartphone [37]. GSMA report revealed that though women have exposure to mobile phones, the percentage of women having exposure to mobile internet is significantly lesser than men [37]. The 75th round of NSSO revealed that 13% of rural and 37.1% of urban populations above age 5 years had the ability to use the internet [36].

In India, there exists a strong stigma on premarital sex, which may result in serious underreporting of premarital sex, this has also been emphasized in a prior study [38]. One apparent limitation is social desirability bias, which can lead to underreporting of youth’s, particularly women’s, involvement in premarital sexual activities. The Youth in India study revealed that the odds of disclosing premarital sexual activities were higher through an anonymous sealed envelope format than through face-to-face interviews [3, 13]. It should be noted that NFHS data was collected through face-to-face interviews, which elevates the odds of underestimation of premarital sexual activities. Another but insignificant limitation of the study is that in the NFHS data, the only contraception use at first sex asked about was ‘condom use’ for unmarried men and women. We considered the use of condoms as a proxy measure for safe sexual activity, as unmarried youth are more likely to be aware of condoms as a method of avoiding pregnancy and are more likely to use modern contraceptives and majorly condoms. The third limitation of the study is that the data collected in NFHS-5 is a cross-sectional data, therefore, the direct causal relationship between digital exposure and premarital sex or condom use must be drawn cautiously.

### Policy recommendation

It is essential to understand the needs of this section of population who has entered into sexual life before marriage, as these youth are also exposed to the risk of unwanted pregnancy, unsafe abortion, and STIs and STDs, including HIV/AIDS due to deeply entrenched stigma in society about sexual activeness before marriage. It is imperative to establish a balanced approach and create online platforms or applications to offer regulated and authorized sex education to youth through mobile devices and the internet. This study provided evidence that mobile and internet exposure was positively associated with increased use of contraception at first sexual encounters. These findings, combined with the increasing ubiquity of mobile and internet use in India, lead researchers of this study to recommend developing comprehensive and culturally sensitive technology-based interventions for imparting sex education to Indian children and youth. This study revealed that youth aged 15–19, with lower education, rural residence, Scheduled Tribe status, having Christian faith, lower wealth status, southern region residenct, alcohol users, having some work experience, living away from home, and limited media exposure are least likely to use condoms during their first premarital sexual encounter. Thus, beyond the central sex education program, these findings underscore the need for other targeted interventions. Family, community and health care institution level interventions are recommended to provide a healthier and stigma-free environment to youth at risk of unprotected premarital sexual activities. In order to deal with the barrier in imparting sex education due to stigma, digital sex education is recommended. Digital sex education makes it accessible, especially in regions where formal sex education is limited or stigmatized. Privacy and anonymity are maintained through it, which is a constant fear in people’s minds due to stigma and adaptability and customization.

Additionally, researchers of this study express a need for more small-scale and in-depth surveys and qualitative studies for premarital sexuality and contraception use to understand the problem more comprehensively instead of relying on large-scale surveys for sensitive issues such as the National Family Health Survey. Application of a combination of data collection techniques such as anonymous surveys, Computer Assisted Self Interviews (CASI), and content analysis of social media data is recommended to increase the validity and reliability of the data on premarital sex and contraception use among unmarried youth.

## Conclusion

Despite the prevailing stigma and taboo surrounding premarital sexual activities, a considerable percentage of women and men were involved in sex before marriage in India. However, the adoption of condoms in premarital sexual activities was not ubiquitous. This study produced evidence that though digital exposure, such as exposure to mobile and exposure to the internet, acted as an influencing factor to increase the youth’s involvement in premarital sexual activities, they also acted as a protective factor for having protected first sexual encounters. Based on these findings, coupled with the escalating adoption of mobile devices and internet usage in India, the implementation of holistic and culturally appropriate technology-driven interventions to provide sex education in India should be practiced.

## Electronic supplementary material

Below is the link to the electronic supplementary material.

Supplementary Material 1

## Data Availability

This study is based on anonymous public-use datasets with no identifiable information about the survey participants. Survey data is available upon request on the official website of the institute at: https://rchiips.org/nfhs/data1.shtml.

## Abbreviations

AIDS: Acquired immunodeficiency syndrome
ATE: Average Treatment Effect
ATT: Average treatment effect among treated
ATU: Average treatment effect on untreated
BSS: National Behavioural Surveillance Survey
CASI: Computer Assisted Self Interviews
CI: Confidence Interval
DHS: Demographic and Health surveys
HIV: Human immunodeficiency virus
NFHS: National Family Health Survey
OBC: Other Backward Caste
PSM: Propensity Score Matching
SC: Scheduled Caste
SDG: Sustainable Development Goals
SMS: Short Message Service
ST: Scheduled Tribe
STD: Sexually Transmitted Diseases
STI: Sexually Transmitted Infections
UDAYA: Understanding the Lives of Adolescents and Young Adults
