# Supplementary material for: Impact of digital exposure on premarital sex and contraception use among unmarried Indian youth
Source: Contracept Reprod Med. 2025 Jan 8;10:3. doi: 10.1186/s40834-024-00334-3 (PMC11708083; doi:10.1186/s40834-024-00334-3)
Supplement: Supplementary file 1 — Supplementary Material 1 [file 40834_2024_334_MOESM1_ESM.docx]

**Title: Impact of Digital Exposure on Premarital Sex and Contraception Use among Unmarried Indian Youths**

**Appendix**

**Table 6. Logistic regression for estimating the association between individuals’ use of mobile phones and their background characteristics, NFHS-5, 2019-21**

| **Owns a mobile telephone/Background characteristic** | **Women** | | **Men** | | |
| --- | --- | --- | --- | --- | --- |
| **Age** |  |  | |  |  |
| 15-19 (ref.) | 1 | [1, 1] | | 1 | [1, 1] |
| 20-24 | 2.41*** | [2.24, 2.59] | | 3.24*** | [2.9, 3.62] |
| 25-29 | 3.74*** | [3.3, 4.24] | | 3.66*** | [3.1, 4.34] |
| **Highest level of education** |  |  | |  |  |
| No education (ref.) | 1 | [1, 1] | | 1 | [1, 1] |
| Primary | 1.23 | [0.96, 1.57] | | 1.03 | [0.84, 1.27] |
| Secondary | 1.55*** | [1.28, 1.87] | | 1.61*** | [1.37, 1.89] |
| Higher | 4.52*** | [3.7, 5.53] | | 9.01*** | [6.89, 11.77] |
| **Place of residence** |  |  | |  |  |
| Urban (ref.) | 1 | [1, 1] | | 1 | [1, 1] |
| Rural | 0.67*** | [0.63, 0.73] | | 0.85*** | [0.77, 0.94] |
| **Caste** |  |  | |  |  |
| SC (ref.) | 1 | [1, 1] | | 1 | [1, 1] |
| ST | 1.80*** | [1.61, 2] | | 0.94 | [0.83, 1.08] |
| OBC | 1.08* | [0.99, 1.18] | | 1.03 | [0.93, 1.14] |
| None of the above | 1.50*** | [1.37, 1.66] | | 1.03 | [0.91, 1.17] |
| **Religion** |  |  | |  |  |
| Hindu (ref.) | 1 | [1, 1] | | 1 | [1, 1] |
| Muslim | 0.91** | [0.83, 0.99] | | 1.31*** | [1.15, 1.5] |
| Christian | 1.29*** | [1.12, 1.48] | | 0.87 | [0.71, 1.05] |
| Others | 1.06 | [0.92, 1.21] | | 1.10 | [0.92, 1.32] |
| **Wealth Index** |  |  | |  |  |
| Poorest (ref.) | 1 | [1, 1] | | 1 | [1, 1] |
| Poorer | 1.11** | [1.01, 1.23] | | 1.27*** | [1.14, 1.42] |
| Middle | 1.47*** | [1.32, 1.63] | | 1.60*** | [1.42, 1.81] |
| Richer | 1.91*** | [1.71, 2.14] | | 1.69*** | [1.47, 1.94] |
| Richest | 2.96*** | [2.61, 3.36] | | 2.31*** | [1.96, 2.72] |
| **Region** |  |  | |  |  |
| North (ref.) | 1 | [1, 1] | | 1 | [1, 1] |
| Central | 0.68*** | [0.62, 0.75] | | 1.16*** | [1.04, 1.29] |
| East | 0.97 | [0.88, 1.08] | | 1.15** | [1.01, 1.32] |
| Northeast | 3.03*** | [2.68, 3.42] | | 1.48*** | [1.25, 1.74] |
| West | 0.88** | [0.78, 0.98] | | 0.93 | [0.78, 1.1] |
| South | 1.11* | [1, 1.24] | | 0.92 | [0.79, 1.07] |
| **Ever worked** |  |  | |  |  |
| Never (ref.) | 1 | [1, 1] | | 1 | [1, 1] |
| Yes | 1.39*** | [1.29, 1.5] | | 3.59*** | [3.28, 3.94] |
| **Stayed away from home** |  |  | |  |  |
| No (ref.) | 1 | [1, 1] | | 1 | [1, 1] |
| Yes | 1.53*** | [1.36, 1.73] | | 1.71*** | [1.5, 1.96] |
| **Bank Account** |  |  | |  |  |
| No (ref.) | 1 | [1, 1] | | 1 | [1, 1] |
| Yes | 3.02*** | [2.81, 3.24] | | 3.11*** | [2.87, 3.37] |
| **Mass media exposure** |  |  | |  |  |
| No Exposure (ref.) | 1 | [1, 1] | | 1 | [1, 1] |
| Partial Exposure | 1.21*** | [1.1, 1.32] | | 1.16*** | [1.05, 1.3] |
| Full Exposure | 1.80*** | [1.6, 2.04] | | 1.67*** | [1.44, 1.94] |
| **Constant** | 0.06 | [0.05, 0.08] | | 0.29 | [0.23, 0.36] |
| **Observations** | **25877** |  | | **27904** |  |

***p<0.01, **p<0.05, *p<0.1; (ref.) represents the reference category
